# Rituximab to treat prolidase deficiency due to a novel pathogenic copy number variation in *PEPD*

**DOI:** 10.1136/rmdopen-2023-003507

**Published:** 2023-12-08

**Authors:** Faranaz Atschekzei, Mykola Fedchenko, Abdulwahab Elsayed, Natalia Dubrowinskaja, Theresa Graalmann, Felix C Ringshausen, Torsten Witte, Georgios Sogkas

**Affiliations:** 1 Rheumatology and Immunology, Hannover Medical School, Hannover, Germany; 2 Cluster of Excellence RESIST (EXC 2155), Hannover Medical School, Hannover, Germany; 3 Institute of Pathology, Hannover Medical School, Hannover, Germany; 4 Junior Research Group for Translational Immunology, TWINCORE, Center for Infection Research and the Hannover Medical School, Hannover, Germany; 5 Biomedical Research in End-Stage and Obstructive Lung Disease Hannover (BREATH), German Center for Lung Research (DZL), Hannover, Germany; 6 Department of Respiratory Medicine and Infectious Diseases, Hannover Medical School, Hannover, Germany; 7 European Reference Network on Rare and Complex Respiratory Diseases (ERN-LUNG), Frankfurt, Germany

**Keywords:** Sjogren's Syndrome, Rituximab, Autoimmune Diseases, Immune System Diseases

## Abstract

Prolidase deficiency (PD) is a rare autosomal recessive inborn error of immunity caused by biallelic homozygous or compound heterozygous loss-of-function mutations in *PEPD*, the gene that encodes prolidase. PD typically manifests with variable dysmorphic features, chronic cutaneous ulcers, recurrent infections and autoimmune features, including systemic lupus erythematosus. So far, there is no consensus regarding treatment of PD and its autoimmune manifestations. Here, we present a 28-year-old female patient with PD due to a novel homozygous intragenic deletion in *PEPD*, diagnosed at the age of 6 years and 7 months with an undifferentiated connective tissue disease that, apart from its very early onset, would be consistent with the diagnosis of Sjögren’s syndrome. Steroids and diverse conventional synthetic disease-modifying antirheumatic drugs failed to control PD-associated vasculitis and mucocutaneous ulcerations and led to infectious complications, including cytomegalovirus colitis. Introduction of rituximab (RTX) treatment in this patient led to sustained recession of mucocutaneous ulceration, enabling tapering of steroids. High interleukin-1β (IL-1β) production by this patient’s monocytes, together with the detection of both IL-1β and interleukin-18 (IL-18) in her serum, suggest enhanced inflammasome activation in PD, whereas the therapeutic efficacy of RTX implies a role for CD20 positive B cells in the complex immunopathogenesis of PD.

**WHAT IS ALREADY KNOWN ON THIS TOPIC**

- Prolidase deficiency (PD) is an inborn error of immunity associated with autoimmunity, especially with systemic lupus erythematosus (SLE).

**WHAT THIS STUDY ADDS**

- We report the second large scale deletion in *PEPD* gene, expanding the genetic spectrum of PD.
- It suggests the therapeutic efficacy of rituximab in treating PD-associated autoimmunity.

**HOW THIS STUDY MIGHT AFFECT RESEARCH, PRACTICE OR POLICY**

- This study may raise awareness among rheumatologists of PD and lead to its consideration in case of patients with dysmorphic features and early-onset autoimmunity mimicking SLE or Sjögren’s syndrome.
- Early consideration of rituximab treatment in patients with PD may spare infectious and autoimmune complications.

## Introduction

Prolidase or peptidase D (PEPD) is a cytosolic metalloproteinase hydrolysing dipeptides with a C-terminal proline or hydroxyproline.^1^ Proline and hydroxyproline are ubiquitous collagen amino acids, constituting more than 10% of the residues of collagen proteins.^2^ Hence, dipeptides produced during the catabolism of collagen are major prolidase substrates.^1^ Given its involvement in collagen catabolism, prolidase plays an essential role in extracellular matrix remodelling and consequently in wound healing and inflammation.

Biallelic homozygous or compound heterozygous loss-of-function mutations in *PEPD*, the gene encoding prolidase, cause an autosomal recessive inborn error of immunity (IEI), prolidase deficiency (PD), falling under diseases of immune dysregulation and in particular, under the subgroup of autoimmunity.^3 4^ Typical manifestations of PD include dysmorphic features, chronic skin ulcers, recurrent infections and features of autoimmune connective tissue diseases. Associated laboratory findings include thrombocytopaenia, hypergammaglobulinaemia, detection of diverse autoantibodies and hypocomplementaemia. So far, 92 cases and 35 pathogenic mutant alleles have been reported worldwide.^3 5^ Diagnosis of PD is based on the identification of high excretion of imidodipeptides in urine or the reduced enzymatic activity of prolidase in erythrocytes and leukocytes in patients with characteristic clinical features, which leads to genetic testing for *PEPD* mutations.

Here, we report a 28-year-old female with PD, displaying features of early-onset Sjögren’s syndrome (SjS) and vasculitis, due to a novel homozygous large deletion in *PEPD*. Rituximab (RTX) treatment in this patient was successful in controlling vasculitis and cutaneous ulcerations.

### Case presentation

**Figure 1:**
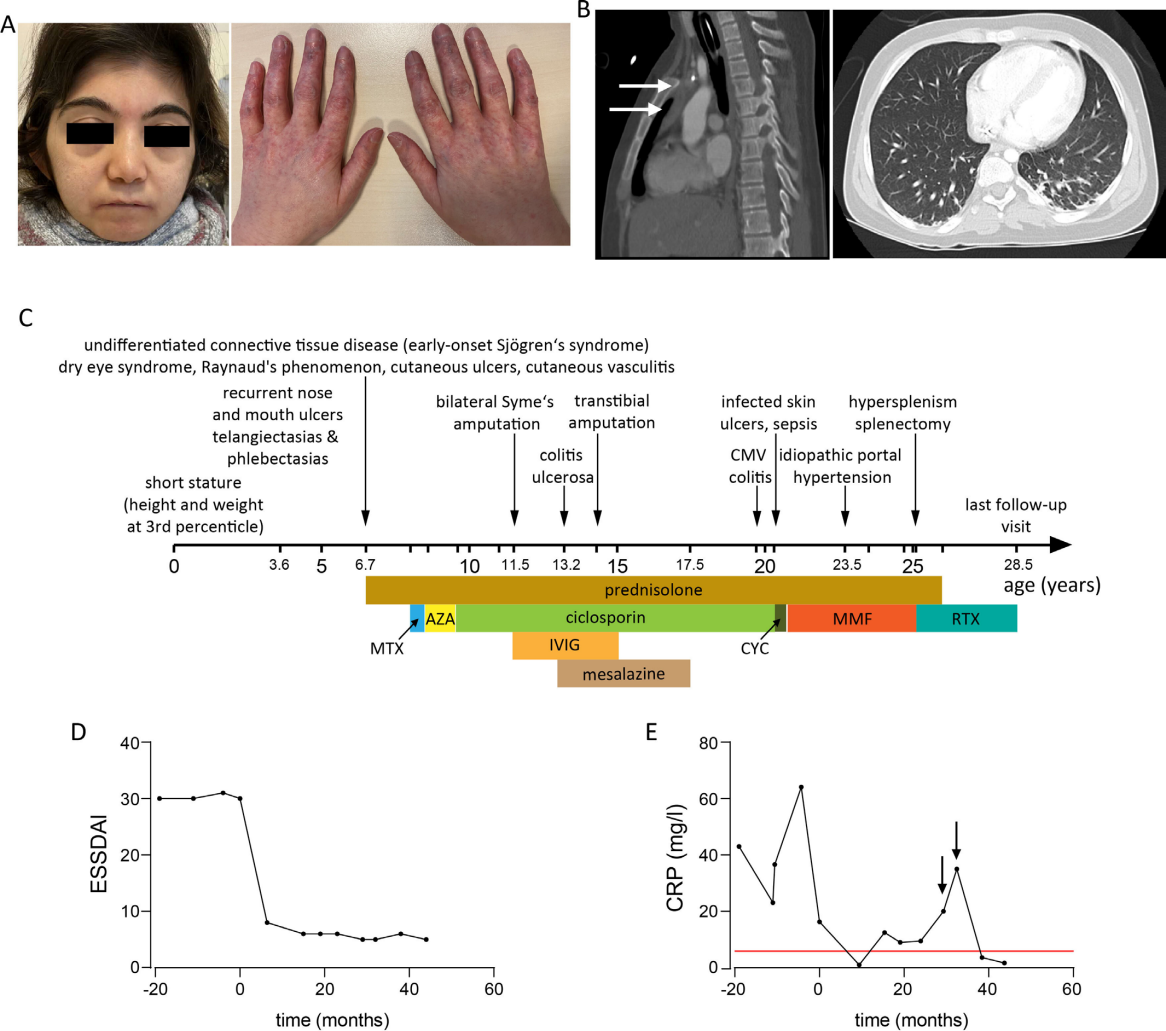
Low hairline, micrognathia and symmetric telangiectasias at fingers and hands of a patient with prolidase deficiency (PD) (A). CT findings. CT of the lungs showing thymic hyperplasia (indicated with arrows) and bilateral basilar bronchiectasis (B). Timeline depicting the course of PD in studied patient together with employed immunomodulatory treatments (C). Course of European Alliance of Associations for Rheumatology (EULAR) Sjögren’s syndrome (SjS) Disease Activity Index (ESSDAI) prior and during treatment with rituximab (RTX) (D). Course of C reactive protein (CRP) serum values prior and during treatment with rituximab (RTX). Arrows indicate peaks associating with the diagnosis of bronchitis due to *Haemophilus influenza* (E; red line highlights upper limit of reference range).

**Figure 2:**
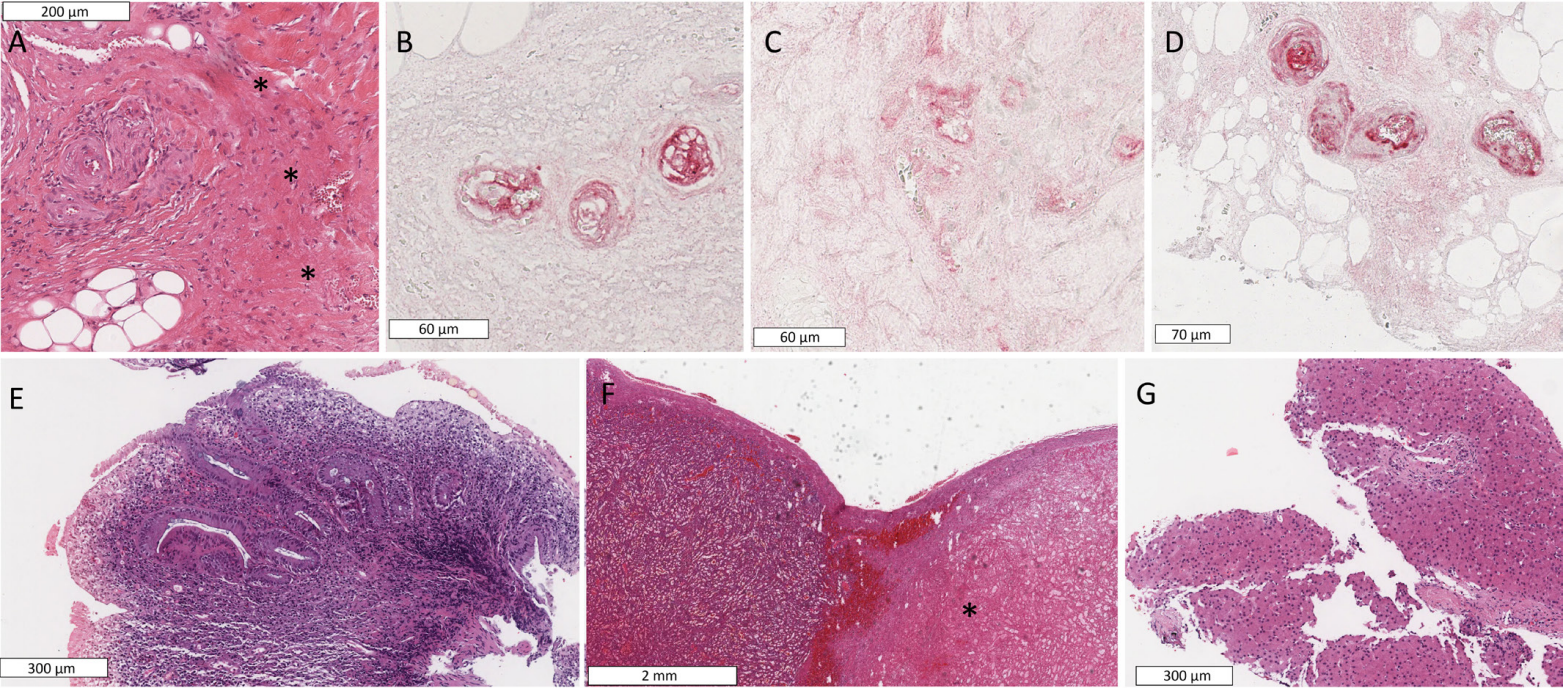
Histopathological findings from a patient with prolidase deficiency. Biopsy of nasal septal mucosa revealing vascular occlusion with leukocytoclasia and fibrinoid vessel wall necrosis (marked with *) (A) and immunohistochemistry of same biopsy revealing substantial C1q, C3 and IgM deposition at the wall of inflamed arterioles (B–D, respectively). Rectum biopsy (E), revealing chronic active ulcerating colitis. Splenic section after splenectomy, revealing an infarction of unknown aetiology (marked with *) (F). Liver biopsy (G), showing ectasia of small portal vein branches and adjoining sinusoids, and minimal lobular and portal hepatitis.

The index patient (figure 1A) is the older one of two female siblings born to healthy non-consanguineous parents of Turkish descent. She displayed dysmorphic features, including a low hairline, a depressed nasal root and micrognathia and telangiectasia predominantly at her hands and feet. Since the age of 3 years and 7 months, she displayed painful mucocutaneous ulcers in her mouth, nose and feet. In addition, she displayed focal painful parchment-like skin lesions at both her feet. She had a history of recurrent bronchitis since the age of 2 years. At the age of 6.7 years, she was diagnosed with an undifferentiated connective tissue disease, whose diagnostic workup revealed fulfilled classification criteria of SjS (SjS), though very early disease onset was not typical for primary SjS.^6 7^ This diagnosis was made on the basis of clinical findings, including bilateral keratoconjunctivitis sicca with positive Schirmer’s test, Raynaud’s phenomenon and cutaneous vasculitis, histopathological findings confirming vasculitic aetiology of mucosal ulcerations (figure 2A–D) as well as laboratory findings and in particular, polyclonal hypergammaglobulinaemia, hypocomplementeamia and antinuclear antibodies with positive Ro (SS-A) and La (SS-B) antibodies. Due to aforementioned diagnosis, prednisolone treatment was started, initially as intravenous pulse treatment with a dose of 10 mg/kg and combined with a variety of conventional synthetic disease-modifying antirheumatic drugs (csDMARDs) (methotrexate, azathioprine, ciclosporin). Aggravation of ulcerations, especially at patient’s feet, led also to high-dose immunoglobulin treatment. All aforementioned treatments failed to control chronic ulcers, which led to a bilateral Syme’s amputation at the age of 11 years and 6 months, a transtibial amputation at the age of 14 years and repetitive wound debridement thereafter. At the age of 13 years, the patient presented with chronic diarrhoea and was diagnosed with colitis ulcerosa. Besides infected skin ulcers and surgical wounds, the patient displayed no discernible infections during her middle and late childhood. However, at the age of 19 years she was diagnosed with cytomegalovirus colitis that was attributed to the immunosuppressive effect of ciclosporin and prednisolone treatment. A CT scan of the lungs revealed bilateral basilar bronchiectasis, when she was 20 years old (figure 1B). Same CT scan showed a thymus hyperplasia. An infection history and the latter finding led to immunological investigations that revealed reduced T-cells and especially CD4^+^-T cell counts (online supplemental table S1), a finding that led to cotrimoxazole prophylaxis. At the age of 23 years, she displayed ascites and splenomegaly and was diagnosed with portal hypertension, whose aetiology remains unknown. Shortly thereafter, she developed pancytopenia attributed to hypersplenism, which led to splenectomy (figure 2F) and consequently improvement of pancytopenia. Given the refractory course of cutaneous ulcerations and pathological evidence suggesting their vasculitic aetiology, an RTX treatment was initiated at the age of 25 years. This included initially two intravenous 1 g infusions separated by 2 weeks, followed by single 1 g infusions every 6 months. Since the introduction of RTX, chronic mucocutaneous ulcerations resolved completely and C3 as well as C4 complement values remained normal. These findings associated with a sustained reduction in the European Alliance of Associations for Rheumatology SjS Disease Activity Index (figure 1D).^8^ Within the 44-month follow-up period since the introduction of RTX no new mucocutaneous ulcerations appeared. She further displayed no additional opportunistic or other severe infections. During the aforementioned follow-up period, only three upper respiratory tract infections (detection of *Haemophilus influenzae* in sputum tests in two of those) have been documented (figure 1E). A chronological summary of the disease course and immunomodulatory medication of the patient is shown in figure 1C.

10.1136/rmdopen-2023-003507.supp1 Supplementary data

**Figure 3:**
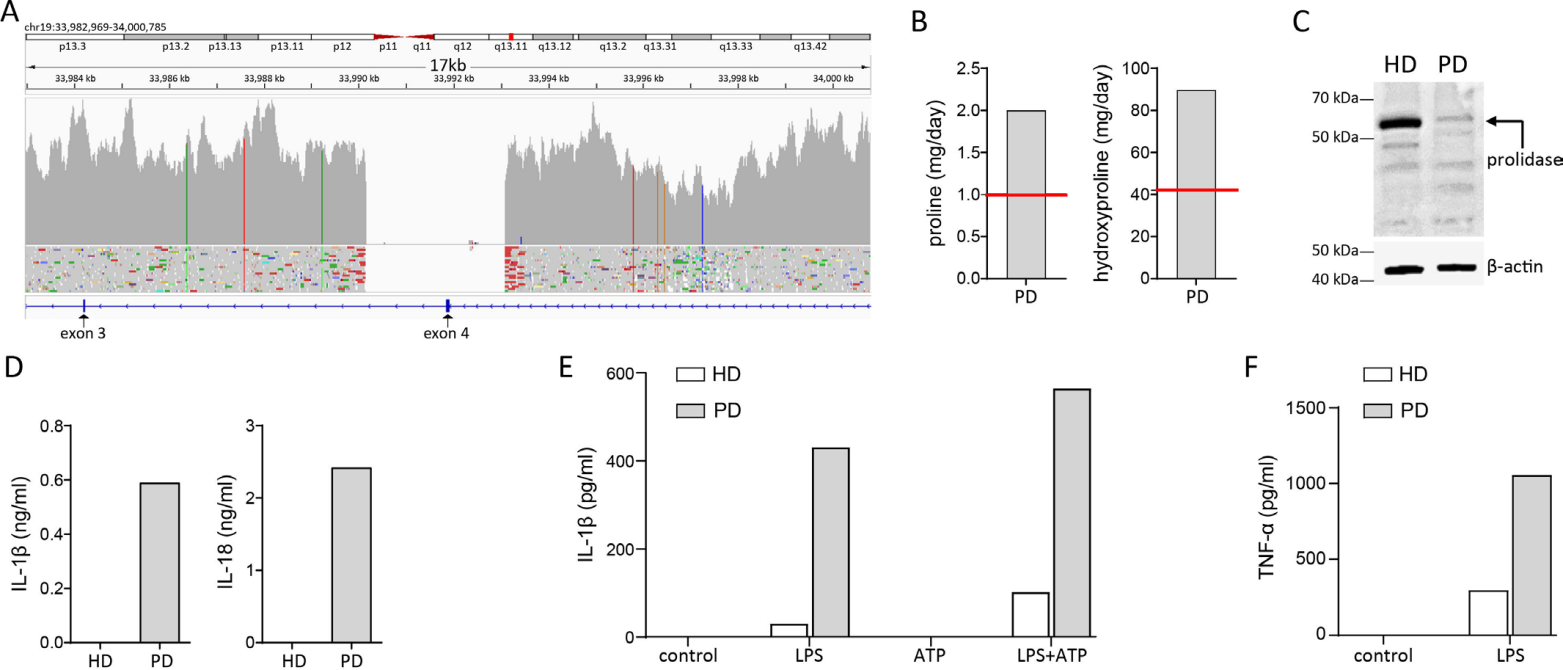
Integrative genomics viewer (IGV) screenshot from whole genome sequencing (WGS) analysis of the studied patient, showing the large 3 kb deletion in *PEPD*, spanning exon 4 (A). Western blotting performed with peripheral blood mononuclear cell (PBMC)-derived protein showing the loss of prolidase expression (B). Urine proline and hydroxyproline levels measured in a 24-hour urine collection (C; red line highlights upper limit of reference range). High serum levels of interleukin-1β (IL-1β) and interleukin-18 (IL-18) in studied PD patient compared with a healthy blood donor (HD). IL-1β and IL-18 were measured with standard ELISAs (D). Higher IL-1β secretion by monocytes from studied PD-patient, compared with an HD, stimulated with lipopolysaccharide (LPS; 500 ng/mL) and (ATP; 1 mM) (E). Finally, enhanced production of tumour necrosis factor α (TNF-α) by monocytes from studied PD-patient, compared with an HD, stimulated with ultrapure lipopolysaccharide (LPS; 500 ng/mL) (F).

Dysmorphic features, the early onset of autoimmunity and the multiple affected organs suggested an underlying IEI. Therefore, we initiated genetic testing, by means of targeted next-generation sequencing, aiming at evaluating the diagnosis of an autoinflammatory disorder and in particular, a type I interferonopathy, as performed previously,^9^ which yielded no pathogenic variant. Thereafter, we performed whole genome sequencing (WGS), which revealed a homozygous intragenic deletion of approximately 3 kb in *PEPD* gene, spanning exon 4 (NC_000019.10:g.(33989982_33992982del); (33989982_33992982del)) (figure 3). To confirm the diagnosis of PD we initiated a 24-hour urine collection, which led to detection of high urinary excretion of proline and hydroxyproline, consistent with a PD-associated imidopeptiduria. Western blotting of protein from the patient’s peripheral blood mononuclear cell (PBMC) revealed the absence of prolidase expression. Consistent with previous reports, the present patient displayed elevated serum levels of IL-18. In addition, we detected elevated serum levels of IL-1β. Evaluation of IL-1β- and IL-18 in serum has been only performed at last follow-up visit, after introduction of RTX treatment and was suggestive of enhanced inflammasome activation, that however, did not correlate with clinically evident disease activity (figure 1D). To evaluate the latter, monocytes were isolated from the patient’s PBMC and stimulated with LPS-ATP. Indeed, patient-derived monocytes displayed higher IL-1β secretion as measured by ELISA (figure 3E), that was already present after stimulation with LPS only.

## Discussion

Here, we report a case of PD due to a novel large copy number variation (CNV), spanning exon 4 of *PEPD*, identified through WGS. With the exception of a previously reported large deletion in *PEPD*,^10^ all so far reported pathogenic variants in *PEPD* were small scale ones.^1^ Considering aforementioned novel variant, 36 pathogenic variants have been reported to cause PD. Those include 16 missense/nonsense variants, 9 indels, 9 splice variants and 2 CNVs.

Autoimmunity in PD can manifest as systemic lupus erythematosus (SLE)-like disease in approximately 10% of patients ranging from typical serological evidence of SLE to severe manifestations such as nephritis and vasculitis.^1 3 5^ Additional immune-related manifestations include vasculitis, AIHA, dermatitis and arthritis. Very early SjS or fulfilment of the relevant diagnostic criteria^6^ in the present patient, expands the phenotypic spectrum of PD. The exact mechanism of autoimmunity in PD remains unclear.^1^ Thymic hyperplasia in the present patient may indicate a defect in central tolerance.^11^ Consistent with previous reports,^3 5^ the present patient displayed elevated serum levels of IL-18, suggesting increased inflammasome activation, which was confirmed by the higher LPS and ATP-induced IL-1β secretion by patient-derived monocytes. The efficacy of RTX in controlling PD-associated autoimmunity suggests the pathogenic relevance of autoantibodies and new plasma cell differentiation (as targeted CD20^+^ B cells are required intermediary cells) or the pathogenicity of alternative B cell functions other than the production of autoantibodies, such as their antigen-presenting role.^12 13^

Efforts to treat PD with replacement of prolidase activity included blood transfusions, gene therapy with an adenoviral vector and enzyme replacement with liposome-coated prolidase.^3 5^ All those approaches were of limited efficacy.^3^ Allogenic hematopoietic stem cell transplantation (HSCT) has been tried in a single patient, who despite reconstitution of prolidase activity died 3 months after HSCT of an invasive fungal infection.^14^ In this case, steroids, diverse csDMARDs and high-dose intravenous immunologlobulin treatment were unsuccessful in treating PD and associated immune dysregulation. Remission of vasculitis and consequently steroid tapering were only possible after the introduction of RTX treatment. Consistent with a previous report by Sato *et al*, reporting the efficacy of RTX as an induction treatment for lupus nephritis and skin ulcers in a 16-year-old male with PD,^15^ here we report sustained regression of vasculitis and mucocutaneous ulcers in a patient with PD. In case of presented patients, csDMARDs were prioritised and repetitive surgical interventions and infectious complications led to a relatively late introduction of RTX. This case together with the report by Sato *et al* suggest the early consideration of RTX treatment in patients with PD displaying autoimmunity.

Identification of IEIs among patients with well-classifiable rheumatic disorders can be a clinically relevant diagnostic challenge for rheumatologists.^16^ Red flags suggesting an underlying IEI in rheumatic patients include infectious complications, persistent secondary hypogammaglobulinaemia and secondary haemophagocytic lymphohistiocytosis or macrophage activation syndrome.^16–19^ In the present case and overall in PD, early-onset treatment-refractory connective tissue disease associating with dysmorphic features should lead to the diagnostic consideration of PD. In addition, chronic typically very painful ulcers and atrophic stellate scars, especially on the feet (atrophie blanche) are very suggestive for this rare disorder.^5 20^ Raising awareness of PD and other rare IEIs, especially disorders of immune dysregulation whose clinical spectrum overlaps with rheumatic conditions, may lead to timely genetic diagnosis and improved clinical outcomes.
