# Supplementary material for: Rituximab to treat prolidase deficiency due to a novel pathogenic copy number variation in PEPD
Source: RMD Open. 2023 Dec 8;9(4):e003507. doi: 10.1136/rmdopen-2023-003507 (PMC10711922; doi:10.1136/rmdopen-2023-003507)
Supplement: Supplementary data [file rmdopen-2023-003507supp001.pdf]

Table S1: Immunological investigations of a patient with prolidase deficiency, performed during treatment with mycophenolate mofetil

Parameter		normal range
**Lymphocytes**		
Lymphocytes (% leukocytes)	18	20-44
Lymphocyte count (cells/µl)	667	1100-4500
CD3+ T cells (% lymphocytes)	58	55-83
CD4+ T cells (% lymphocytes)	28	28-57
CD8+ T cells (% lymphocytes)	36	10-39
CD19+ B cells (% lymphocytes)	19	6-19
CD3+CD56+ NK cells (% lymphocytes)	11	7-31
Gamma/delta T cells (% CD3+ T cells)	8	<15%
Alpha/beta T cells (% CD3+ T cells)	93	>85%
**Immunoglobulins**		
IgG (g/l)	32.7	7-16
IgA (g/l)	6.7	0.7-4
IgM (g/l)	1.1	0.4-2.3
Meales IgG antibody (result/ U/l)	positive/16000	n.a./> 13000
Meales IgM antibody (result)	negative	n.a.
EBV IgG antibody (result)	positive	n.a.
EBV IgM antibody (result)	negative	n.a.
CMV IgG antibody (result)	positive	n.a.
CMV IgM antibody (result)	negative	n.a.
**Lymphocyte proliferation assay**		
Phytohaemagglutinin (% control)	88.3	100
Concanavalin A (% control)	87.8	100
Pokeweed mitogen (% control)	146	100
Tuberculin purified protein derivative (% control)	66	100
Interleukin-2 (% control)	175	100
Anti-CD3-antibody (% control)	133	100
**NK cell function**		
natural cellular cytotoxicity (% control)	160	100
antibody-dependent cellular cytotoxicity (% control)	92.5	100
